# Small-area spatial statistical analysis of malaria clusters and hotspots in Cameroon;2000–2015

**DOI:** 10.1186/s12879-018-3534-6

**Published:** 2018-12-07

**Authors:** Marlvin Anemey Tewara, Prisca Ngetemalah Mbah-Fongkimeh, Alimu Dayimu, Fengling Kang, Fuzhong Xue

**Affiliations:** 1 Department of Epidemiology and Biostatistics, School of Public Health, Shandong University Cheeloo College of Medicine , Jinan, 250012 People’s Republic of China; 2 School of Medicine and Public Health, Faculty of Health and Medicine, The University of New Castle, Callaghan, Australia

**Keywords:** Spatial statistics, Urban-rural, Epidemiology, Hotspots, Clusters, Malaria, Mapping

## Abstract

**Background:**

Malaria prevalence in Cameroon is a major public health problem both at the regional and urban-rural geographic scale. In 2016, an estimated 1.6 million confirmed cases, and 18,738 cases were reported in health facilities and communities respectively, with about 8000 estimated deaths. Several studies have estimated malaria prevalence in Cameroon using the analytical techniques at the regional scale. We aimed at identifying malaria clusters and hotspots at the urban-rural geographic scale from the Demographic and Health Survey (DHS) data for households between 2000 and 2015 using ArcGIS for intervention programs.

**Methods:**

To identify malaria hotspots and analyze the pattern of distribution, we used the optimized hotspots toolset and spatial autocorrelation respectively in ArcGIS 10.3 for desktop. We also used Pearson’s Correlation analysis to identify associative environmental factors using the R-software 3.4.1.

**Results:**

The spatial distribution of malaria showed statistically significant clustered pattern for the year 2000 and 2015 with Moran’s indexes 0.126 (*P* < 0.001) and 0.187 (P < 0.001) respectively. Meanwhile, the years 2005 and 2010 with Moran’s indexes 0.001 (*P* = 0.488) and 0.002 (*P* = 0.318) respectively, had a random malaria distribution pattern. There exist varying degrees of malaria clusters and statistically significant hotspots in the urban-rural areas of the 12 administrative regions. Malaria cases were associated with population density and some environmental covariates; rainfall, enhanced vegetation index and composite lights (*P* < 0.001).

**Conclusion:**

This study identified urban-rural areas with high and low malaria clusters and hotspots. Our maps can be used as supportive tools for effective malaria control and elimination, and investments in malaria programs and research, malaria prevention, diagnosis and treatment, surveillance, should pay more attention to urban-rural geographic scale.

## Background

Malaria remains an international public health challenge as there has been an increase in the number of estimated malaria cases; 5 million malaria cases from 2015 (211 million) to 2016 (216 million) [1]. Worldwide,109 countries are now malaria-free, whereas malaria is still an endemic disease in about 99 countries [2]. About 90 and 91% of malaria cases and deaths respectively, reported in 2016 occurred in the WHO Africa region with about 15 counties all in Sub-Saharan Africa (SSA) [1]. The most prevalent malaria parasite in SSA is the *Plasmodium falciparum*, accounting for 99% of malaria cases and most occurring in children under the age of five [1]. In Cameroon, the epidemiological transmission of malaria is high (> 1 case per 1000 population) in about 71% (16.6 million people) and low (0–1 cases per 1000 population) in about 29% (6.8 million) in people of all sexes and age groups. Malaria prevalence in Cameroon is a major public health problem at both the regional (larger) and urban-rural (smaller) geographic scales, with an estimated 1.6 million confirmed cases reported in health facilities and 18,738 cases at the community level and 8000 (6000-10,000) estimated deaths in 2016 [3]. Generally, malaria intervention policies and control strategies in both the regional and urban-rural scales in Cameroon, have been reported to focus on the use of insecticide treated bed-nets (ITNs), indoor residual spray (IRS),larval control, diagnostic testing, treatments, disease surveillance, and national campaigns [3–6].

The WHO and the roll back malaria global action plan [7] anticipate having a malaria-free world by 2030 through its set milestones and targets pillars with a major focus to ensure universal access to malaria prevention, diagnosis, and treatment. Malaria prevention strategies based on the use of ITNs and or IRS in Cameroon, has been a great method in the reduction of incident cases of the disease as about 13.6 million ITNs deliveries of the 80% ITNs deliveries in SSA, was made in Cameroon between 2014 and 2016 [1].

Malaria risk maps and the applications of spatial malaria epidemiology in the fight against malaria in Africa has been limited. A review by Omumbo [8], examining the most recent national malaria strategies, monitoring and evaluation plans, as well as the types of maps presented and how they have been used to define priorities for investments in malaria control in 47 countries in Africa, found that about 32% of the countries did not present malaria maps within their national malaria prevention strategies.

Small-area statistical analysis and spatial epidemiology have emerged to solve issues of where disease clusters and hotspots are located. Spatial epidemiology deals with the analysis and description of geographic health data with respect to demographic, environmental, behavioral, socioeconomic, genetic and other infectious agents or risk factors [9]. A study by Elliot [9] on the current approaches and future challenges of Spatial epidemiology reported that, recent advancements in data availability and analytical methods have created new openings for studies to improve on the local reporting of diseases at national or regional scale by observing changes in disease prevalence rates at a smaller scale. Although, they reported on the absence of a satisfying definition of the term small-area in studying the variations in disease incidence and mortality, [10] suggested a working definition as a rough guide which we will apply in our study; any region containing fewer than about 20 cases of a disease can be considered a small area. For example, a disease with an annual incidence rate of about 5 per 100, 000 for a period of 5 years, a small area constitutes a population size of around 100, 000 or fewer in clusters of disease occurrence in a remote area or small village. They also identified four types of Spatial analysis at a small-area scale: disease mapping, geographic correlation studies, disease clusters, and surveillance. Some of the main techniques of spatial methods reviewed by Robertson [11], used in emerging infectious disease research include; spatial autocorrelation, space-time interactions, hotspots and clusters.

The global spatial autocorrelation technique is used to characterize a full map in one quantitative value. This method measures the total joint counts of nearby regions, attributes or locations against a null hypothesis of no spatial autocorrelation [11]. Moran’s I and Geary’s *c* statistics are common methods of spatial autocorrelation. Positive spatial autocorrelation indicates the existence of clustered patterns of a disease, negative autocorrelation can suggest a dispersion in the transmission pattern or surveillance among given regions. Hotspot mapping and cluster detection are analyses executed through local spatial analysis methods. The basic technique is to calculate a test statistic for each location and then evaluate the distribution of these test statistics against a theoretical or random reference distribution. This technique is important in infectious diseases surveillance in that, it helps to identify geographic areas where and to what extent an observed spatial pattern of a disease is anticipated relative to a null hypothesis [9, 11].

Smith [12], conducted a systematic review of published reports of outbreak investigations worldwide to estimate the prevalence of infectious diseases using spatial methods such as dot maps, Moran’s I, rate maps, Gestis-Ord Gi* on different diseases; hepatitis, influenza, malaria, rabies and many others. Bhatt [5], found that, *Plasmodium falciparum* infection in endemic Africa has reduced and incidence of the clinical disease fell by 40% between 2000 and 2015; the authors used the Geographical Information Systems (GIS) applications. The GIS computer system can describe, analyze, and predict disease patterns using feature (cartographic) and attribute data. GIS has been used in many epidemiologic applications, including disease mapping, rate smoothing, cluster or hotspot analysis, and spatial modeling and have been reported and applied in small area units such as urban-rural and lower administrative scales [9–13]. Dot maps and geographic profiling have been used both in the United Kingdom and Egypt as spatial methods to identify locations of sources of cholera and malaria infections respectively [14]. The Moran’s I spatial method has also been used to identify cholera clusters in areas with lower coverage of latrines in a peri-urban area of Lusaka, Zambia and advise for effective drainage systems [15]. Moreover, during the 2003 severe acute respiratory syndrome (SARS) outbreak in Hongkong, the Moran’s I technique was used to identify SARS cluster patterns at the community level [16]. Findings from a study carried out in the small-area rural highlands of Western Kenya, identified significant spatial clusters of malaria in school children during an outbreak [17]. The authors used household survey data and their analyses used the spatial scan statistic software.

Most studies focusing on malaria prevalence and incidence, or the use of ITNs / IRS, in Cameroon have applied the analytical statistics methods, tools evaluation, vector control and molecular techniques at both the higher and lower administrative levels [4, 18, 19].

Understanding the distribution of malaria cases in Cameroon with the use of spatial statistical analysis approach, will help inform malaria control programs at a smaller scale. Thus, we aim to identify malaria clusters and hotspots in Cameroon at the urban-rural scale using the DHS Global Positioning System (GPS) data for households. Our objectives are to; i) use the spatial autocorrelation technique to analyze malaria spatial patterns in ArcGIS for desktop, ii) map the distribution of malaria cluster points, iii) identify urban-rural clusters with statistically significant hotspots of the disease, and iv) identify environmental factors associated with the distribution of malaria cases.

## Methods

### Data acquisition

Data for this study was obtained after a granted request for registered users from the DHS program website https://dhsprogram.com funded by the United States Agency for International Developments (USAIDS) MEASURE DHS Project in collaboration with the National Institute of Statistics (Institute Nationale de la Statistique) and the Ministry of Public Health Cameroon. The DHS are nationally-representative, probabilistic, household surveys that include a wide range of key demographic and health indicators used to monitor and evaluate population, health, and nutrition programs [20]. The data contains the Cameroon 2011 DHS malaria data for a five-year (2000, 2005, 2010, and 2015) interval and some environmental covariates; enhanced vegetation index, rainfall, composite lights, and Population density at the urban-rural scale.

### GPS data

The GPS point data for each sampled urban-rural cluster residence was linked to all the households and individual level attributes such as survey information for Malaria Indicator Survey (MIS) and AIDS Indicator Survey to be analyzed with ArcGIS. For reasons of confidentiality, the GPS urban-rural location points were masked [21, 22] and a python script in ArcGIS was used to displace the data within the appropriate administrative boundaries. For small-area administrative units, urban residence clusters were displaced a distance up to 2 km and rural clusters up to 5 km, with a further randomly selected 1% of the rural clusters displaced up to 10 km [20].

### Sample population

**Fig. 1:**
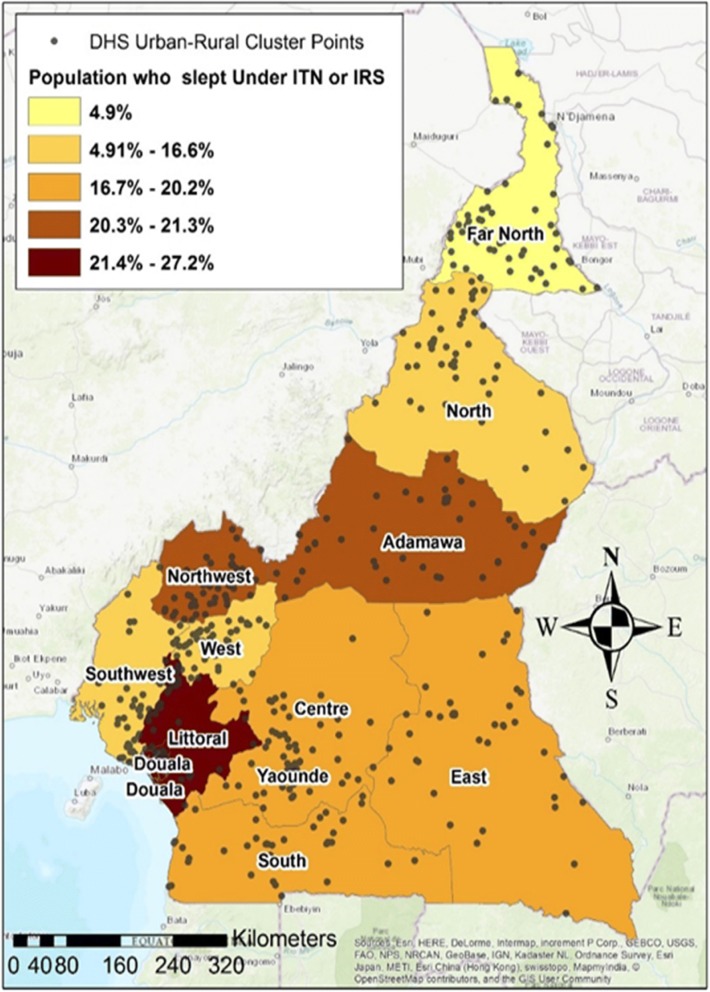
DHS Urban-Rural Cluster point locations (grey dots) and the percentage of people who slept under ITNs and or use IRS the previous night during the 2011 DHS Survey

The DHS urban-rural residence clusters (Fig. 1) as defined by the country’s census bureau, is usually part of the sampling domain for lower levels of administrative units. Census enumeration areas can be a city block or apartment building for urban areas while in rural areas is typically a village or group of villages. The population and size of sample clusters vary between and within countries (Table 1). Generally, clusters contain 100 to 300 households, of which 20 to 30 households are randomly selected for survey participation [20, 23].

**Table 1:** DHS VI National Coverage for the 2011 field work

Major Survey Parameters	Size	Weighted^a^ (Weighted by clusters)
Households	14,214	
Women (15–49 years)	15,426	
Men	7191	
Children < 60 Months	11,732	11,748
Household Members	72,622	72,884
All Births	42,312	42,071
Couples	2973	3003
Urban-Rural GPS Clusters	578	

^a^Weights are adjustment factors applied to each case in the DHS survey to adjust for differences in probability of selection and interview between cases in a sample, due to different study designs [20]

### Malaria data description

A malaria year was described as the average number of people per year who show clinical symptoms of *Plasmodium falciparum* malaria within the cells whose centroid falls within a radius of 10 km (for rural points) or 2 km (for urban points). In surveys that collected specimens for malaria testing (primarily MIS), indicators of the prevalence of malaria are provided, based on both rapid diagnostic tests (RDT) and on laboratory analysis [20]. A clinical case was defined as a malaria-attributable febrile episode (body temperature in excess of 37.5 °C), typically accompanied by headaches, nausea, excess sweating and or fatigue censored by a 30-day window [24, 25].

### Statistical analyses

#### Malaria spatial pattern analysis

The global spatial autocorrelation (Moran’s I) statistical toolsets in ArcGIS10.3 was used to identify statistically significant malaria clusters over the study area for the different regions in Cameroon between 2000 and 2015. The Moran’s Index (M.I) statistical technique, evaluates the spatial autocorrelation of malaria cases at the urban-rural cluster locations where a Moran’s I value close to zero signifies spatial randomness of the disease, a positive value indicates spatial clustering [11, 26]. To evaluate whether the spatial pattern is clustered, dispersed or random, a statistically significant estimate of Moran’s I (*p* < 0.05, z score ≥ 1.96) indicates neighboring urban-rural areas have similar malaria cases under the null hypothesis that the distribution of malaria cases at the urban-rural scale is completely spatially random. The spatial autocorrelation tool runs through an input feature class, a conceptualization of spatial relationship (which include inverse distance, travel time, fixed distance, K nearest neighbors, and contiguity), and a distance band or threshold distance (cases have at least one neighbor). The tool returns five values: the Moran’s I Index, expected index, variance, z-score, and *p*-value. This tool calculates a z-score and p-value which are measures of statistical significance to indicate whether or not the null hypotheses can be rejected.

#### Mapping malaria, INTs / IRS and population density distributions

The average number of people per year who showed clinical symptoms for malaria in the urban-rural residence between 2000 and 2015 was symbolized using graduated symbols with five classes natural breaks Jenks; which is a symbology technique in ArcGIS that shows quantitative differences in data values with varying symbol sizes. The data is classified into ranges that are each then assigned a symbol size to represent the range. The percentage of people who slept under ITNs and or use IRS the previous night during the DHS survey was mapped using the choropleth mapping technique where color shades range from light (lower data values) to dark (higher data values). The average number of people per square kilometer (UN Population density) for the different administrative regions was mapped using the heat map symbology technique. This approach displays the relative density of points using a color scheme, ranging from low density to high density of points.

#### Malaria hotspot analysis

Moran’s I have well established statistical properties to describe global spatial autocorrelations but has not been effective in identifying clustered spatial patterns and hotspots [26]. For hotspots identification, we employed the optimized hotspot analysis tool in ArcGIS10.3 to identify the malaria incident hotspots at the urban-rural clusters. The optimized hotspots analysis creates a map of statistically significant hotspots (areas with high malaria cases) and coldspots (areas with low malaria cases) using the Getis-Ord Gi^*^ statistic; which uses a default count incident points within fishnet polygons as the incident data aggregation method. The software generates polygons and aggregates these points into the polygons. The fishnet polygon technique produces a map of malaria incident cases with similar attribute values and automatically classifies them as coldspots (blue areas) or hotspots (red areas).

#### Correlational analysis

Malariological measures such as those of the environment, have been reported to be associated with malaria prevalence [17, 27, 28]. To understand the association between malaria case distribution and environmental factors in our study, we measure the associations between the five-year interval malaria cases and environmental covariates such as rainfall, enhanced vegetation, and nightlights composite, by applying the Pearson’s product -moment Correlation Coefficient (PCC) denoted by r. The PCC was analyzed using the R-Statistic Software (version 3.4.1). The mathematical computations and the applications of PCC are documented in [29] and was applied in the spatiotemporal distribution and hotspots of hand, foot and mouth disease in Northern Thailand, where the authors found rainfall to be associated with the spread of the disease [26]. The statistically significant result r, measuring the strength of the associations from − 1 (perfectly inverse association) to + 1(perfectly strong association) were obtained.

## Results

### Spatial autocorrelation pattern

**Fig. 2:**
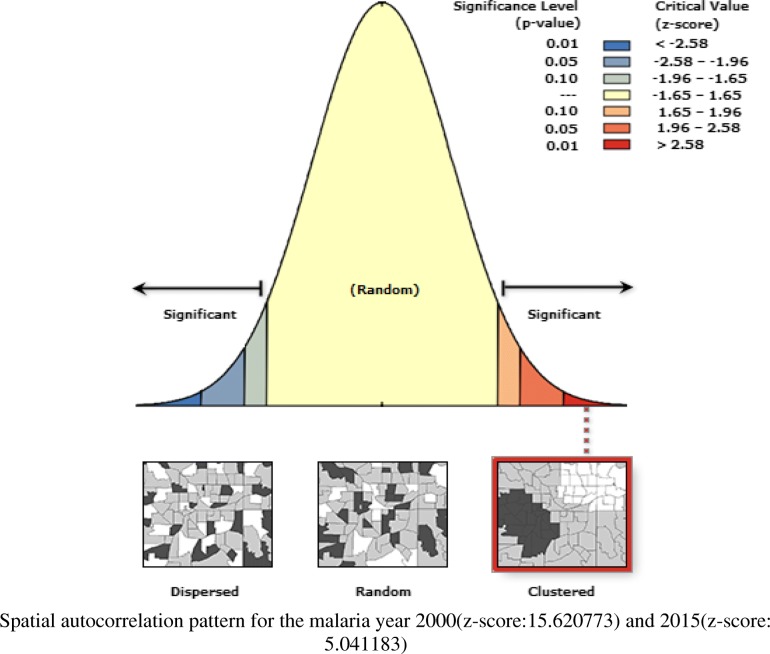
Graph of clustered malaria pattern for the year 2000 and 2015

**Fig. 3:**
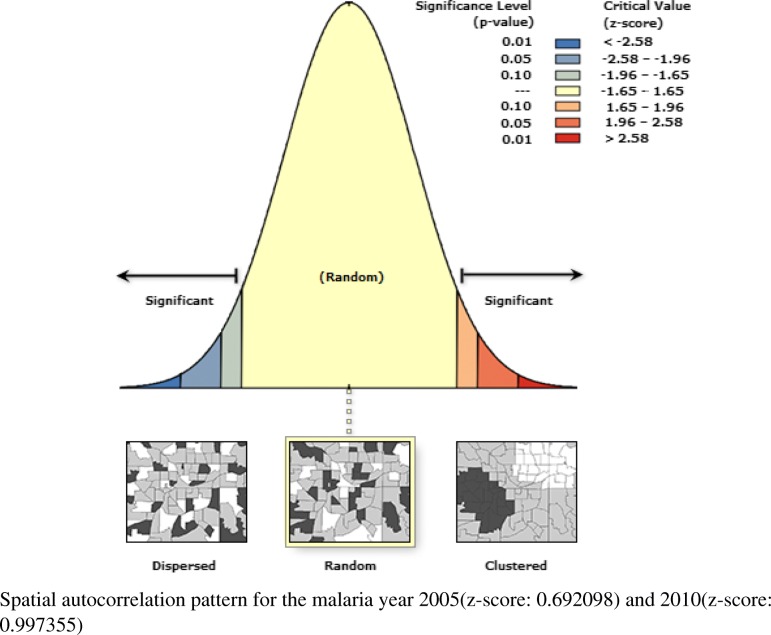
Graph of random malaria pattern for the year 2005 and 2010

The global MI was greater than zero (more positive) for the 2000 and 2015 malaria year (Fig. 2) with Moran’s indexes 0.126(*P* < 0.001) and 0.187(P < 0.001) respectively. Whereas, the year 2005 and 2010(Fig. 3) had values close to zero with Moran’s indexes 0.001(*P* = 0.488) and 0.002 (*P* = 0.318) respectively (Table 2).

**Table 2:** Global Moran’s I summary for the different malaria year

Year	Moran’s Index	Expected Index	Variance	Z-Score	P-value
2000	0.126795	−0.001733	0.000068	15.620773	0.000000
2005	0.001092	−0.002252	0.000023	0.692098	0.488876
2010	0.018808	−0.002252	0.000446	0.997355	0.318592
2015	0.098884	−0.001733	0.000393	5.072826	0.000000

### General malaria distribution

**Fig. 4:**
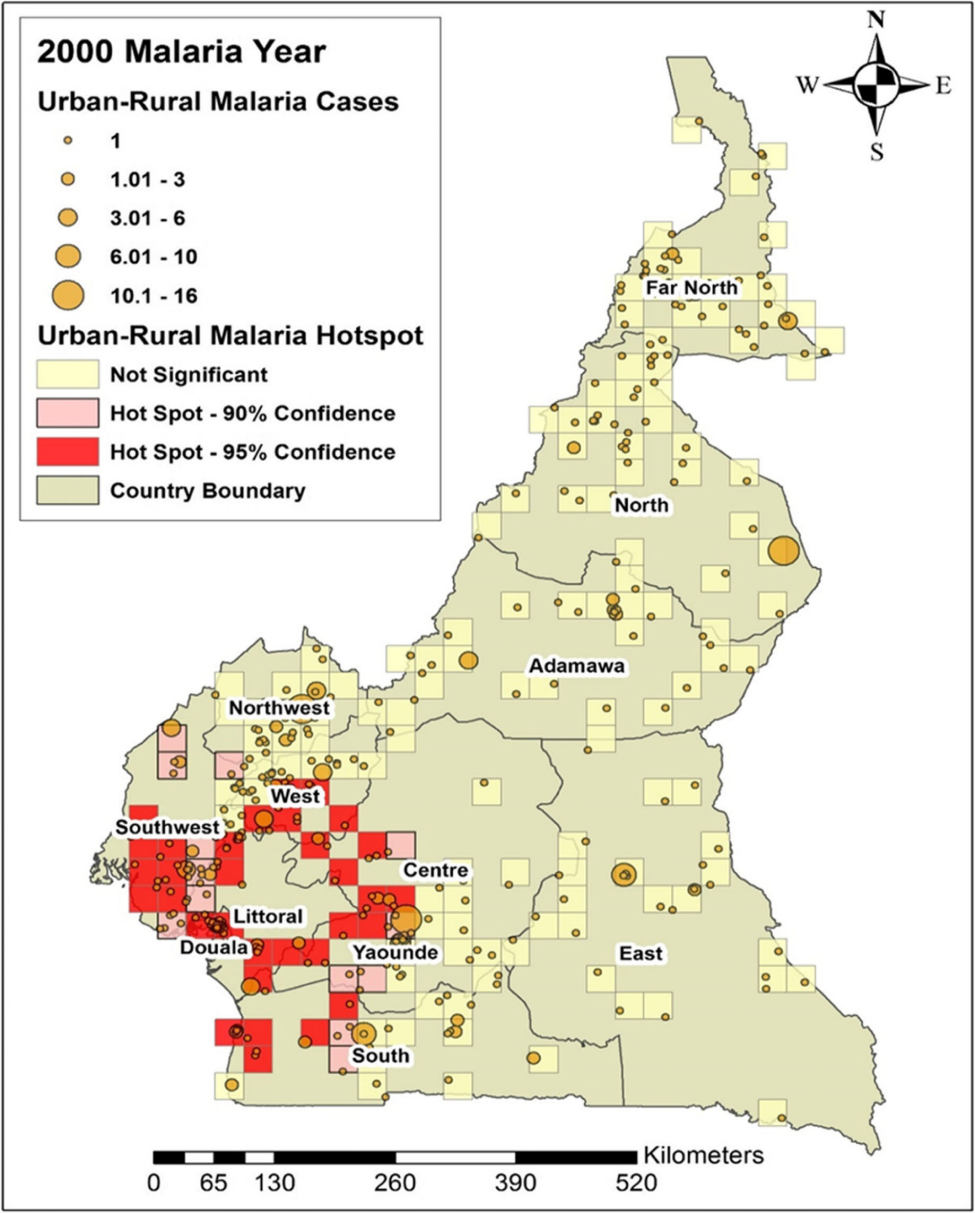
Map of malaria cases (graduated symbol) and statistically significant hotspot locations at the urban-rural clusters for the year 2000

**Fig. 5:**
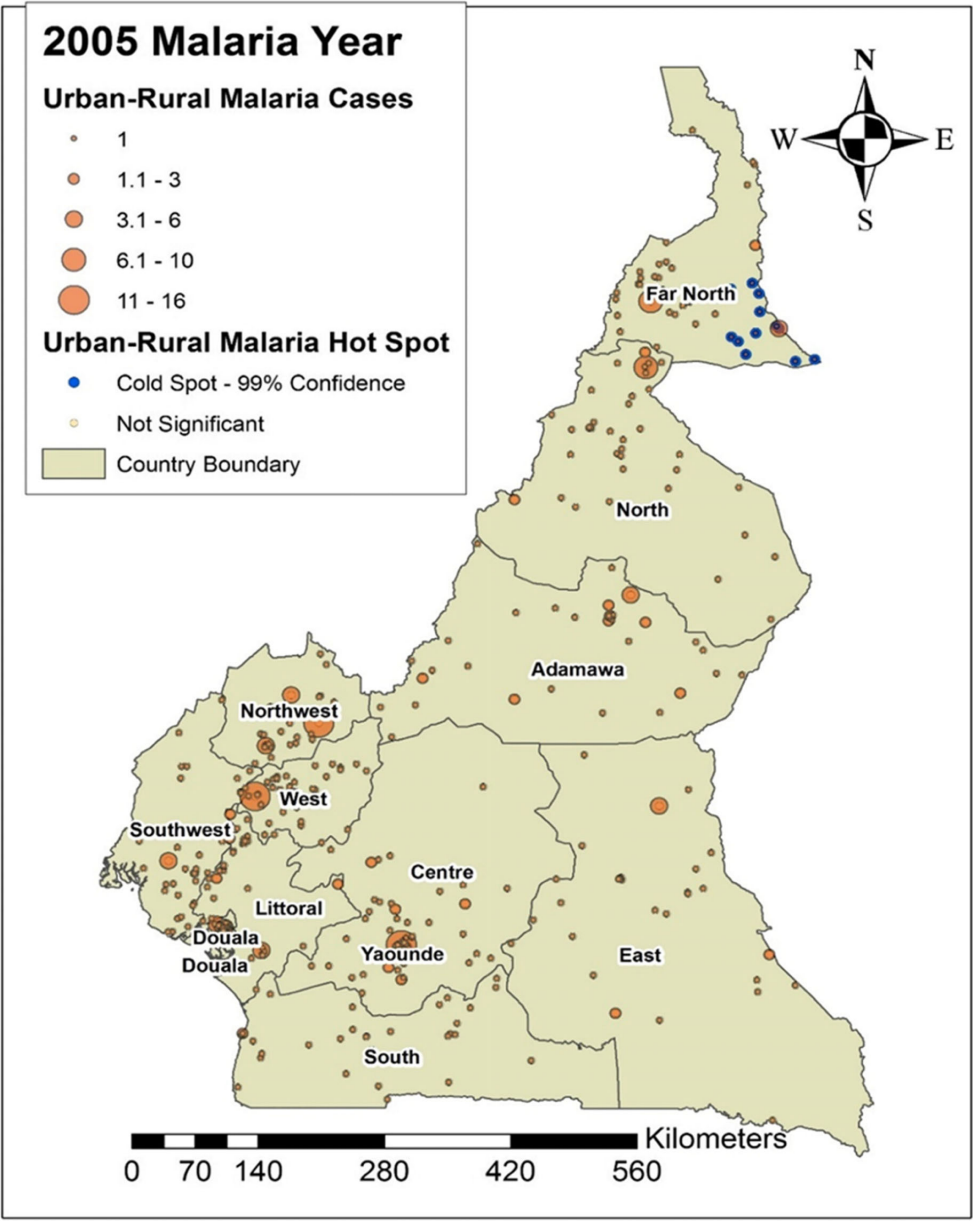
Map of malaria cases (graduated symbol) and statistically significant hotspot locations at the urban-rural clusters for the year 2005

**Fig. 6:**
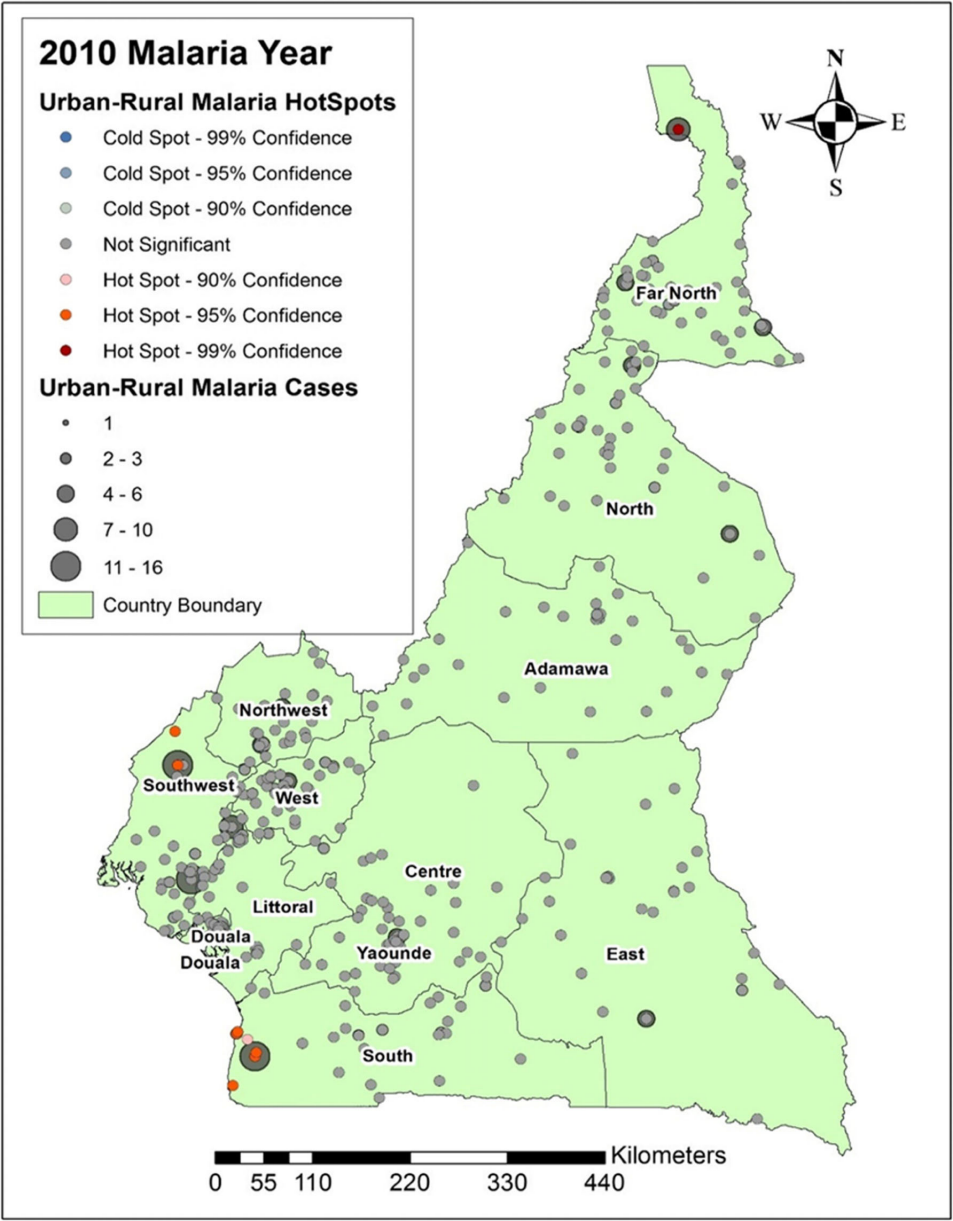
Map of malaria cases (graduated symbol) and statistically significant hotspot locations at the urban-rural clusters for the year 2010

**Fig. 7:**
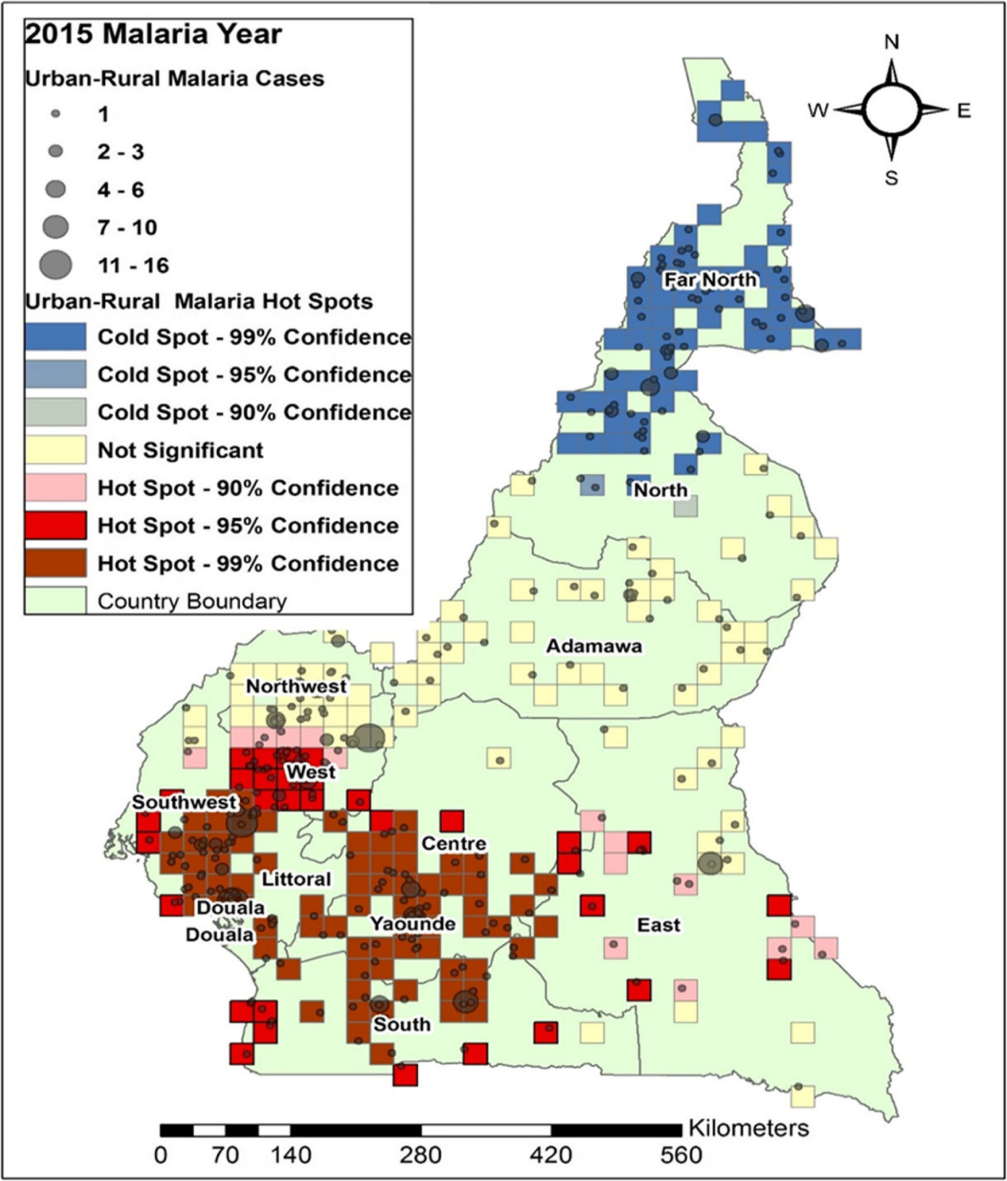
Map of malaria cases (graduated symbol) and statistically significant hotspot locations at the urban-rural clusters for the year 2015

In this study, we looked at the average number of people per year suffering from malaria between the years 2000 and 2015. Figures 4, 5, 6 and 7 shows the distribution of malaria cases by urban-rural clusters in each administrative region. Throughout each of the five-year intervals, 2000, 2005, 2010 and 2015, the highest and lowest urban-rural malaria cases are illustrated with graduated symbols as shown in Figs. 4, 5, 6 and 7.

### Malaria hotspots identification

Our main goal in this study was to identify malaria hotspots locations for future intervention using the spatial autocorrelation statistical techniques. In the year 2000, statistically significant malaria hotspots (95% confidence) were identified in urban-rural clusters of the West, Southwest, Douala, Yaoundé, Littoral, Center and the South regions (Fig. 4). In 2005, there were no statistically significant malaria hotspots in all the 12 DHS administrative regions. The Far north had areas with statistically significant coldspots; areas with low malaria cases (Fig. 5).

In 2010, some urban-rural areas of the Southwest and South regions had statistically significant malaria hotspots (Fig. 6). In 2015, most urban-rural areas in the West, Southwest, Douala, Center, and East had statistically significant malaria hotspots (95% confidence). Some communities in the North had statistically significant malaria coldspots as illustrated in Fig. 7.

**Fig. 8:**
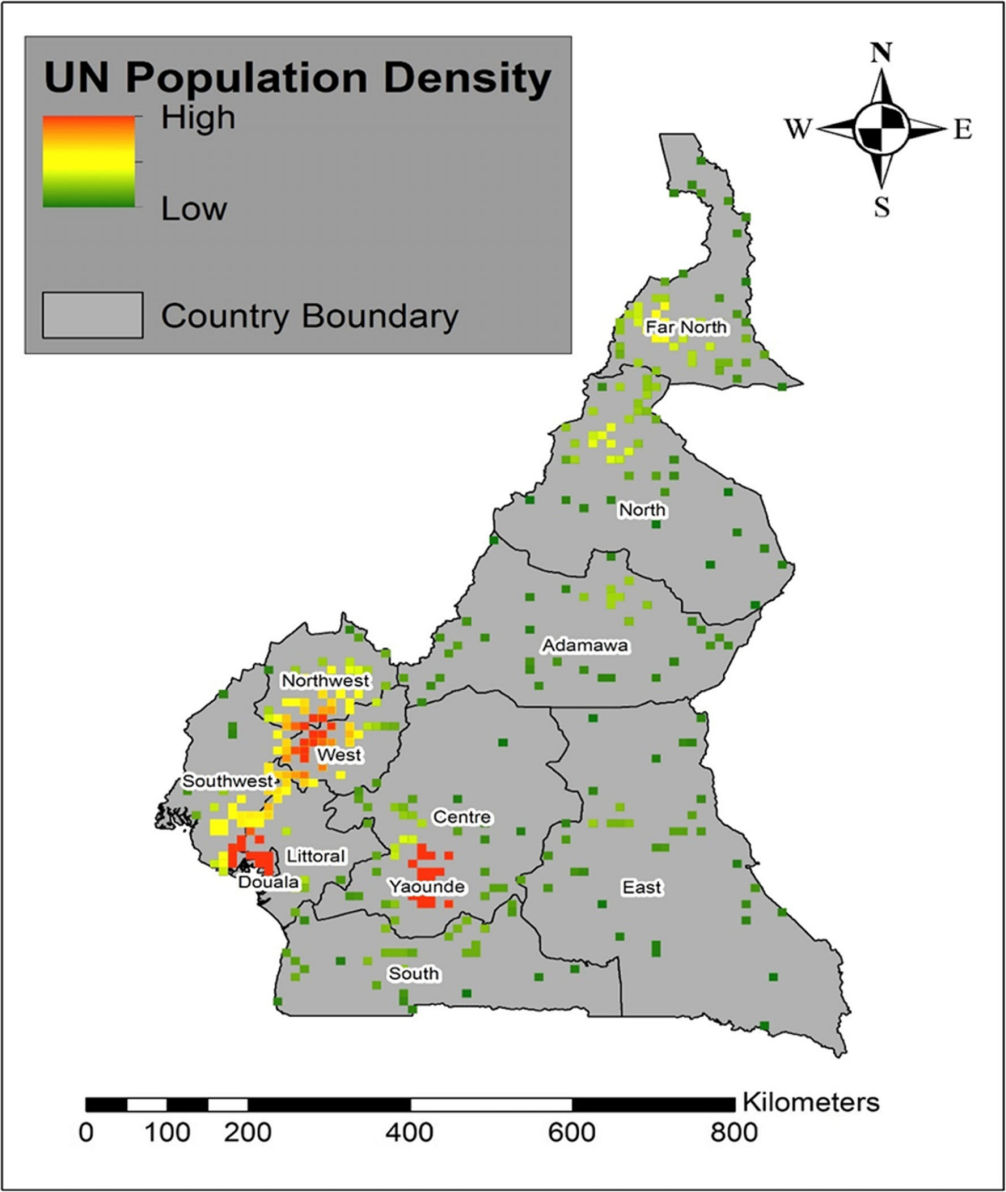
Map showing the Population density at the urban-rural scale for the different DHS administrative regions

The map of the UN population density shows high population densities in the urban-rural clusters of Yaoundé, Douala and West regions and low population densities in some parts of the Southwest, Northwest, North and Far North regions (Fig. 8).

Table 3 demonstrates the strength (r) of the association between the distribution of malaria cases and environmental factors.

**Table 3:** Association between malaria cases and environmental factors based on the strength of the Pearson’s Coefficient (r)

Malaria Year	Environmental Covariate	*t*-value	*p*-value	r
2000	Enhanced Vegetation Index	6.411488736	< 0.001	0.2580944
2005	Enhanced Vegetation Index	6.176010282	< 0.001	0.24921445
2010	Enhanced Vegetation Index	6.099155642	< 0.001	0.24630246
2015	Enhanced Vegetation Index	6.135765971	< 0.001	0.247690448
2000	Rainfall	4.268475912	< 0.001	0.175105281
2005	Rainfall	4.479970031	< 0.001	0.183495927
2010	Rainfall	3.633057011	< 0.001	0.149672211
2015	Rainfall	6.258955928	< 0.001	0.252349658
2015	Nightlights Composite	11.91561684	< 0.001	0.444692469

## Discussion

The application of spatial analytical techniques focusing on malaria is not new. However, very limited studies have focused on smaller administrative levels [17, 30, 31]. Given the z scores: 5.07 and 15.6 for the year 2000 and 2015 respectively, indicate there is a less than 1% likelihood for the observed clustered pattern to be due to chance (Fig. 2). The null hypothesis of complete randomness is rejected, and the presence of cluster patterns indicate neighboring locations have high malaria cases at a given urban-rural area. The z scores: 0.69 and 0.99 for the year 2005 and 2010 respectively, illustrate that the malaria pattern does not appear to be significantly different than random (Fig. 3), and the null hypothesis of complete randomness is accepted. This suggests that malaria cases are randomly spread across the urban-rural areas. Knowing the hotspot locations of areas with clustered malaria patterns can inform for national malaria prevention programs and surveillance.

The distribution of urban-rural malaria cases observed as graduated symbols in Figs. 4, 5, 6 and 7 call for prevention programs as some urban-rural areas in Yaoundé, Douala, Center, South West, North West, Littoral, West, and South region had high malaria cases, and low cases in Adamawa, East, North, and Far North region. Our finding is consistent with that reported by Gemperli [28] where they found high malaria prevalence in the West and low prevalence in the North and Far North. Contrary to our study that focused on the distribution of malaria cases at a smaller scale, the author focused on malaria prevalence at a regional scale. Understanding the distribution of malaria cases and prevalence in these areas will advise for investments and prevention programs.

The hotspots analysis identified varying intensities of malaria hotspots in the urban-rural areas of the West, Southwest, Northwest, Douala, Yaoundé, Littoral, Central, and South regions (95% confidence) between 2000 and 2015(Figs. 4 and 7). In addition, there was a shift in the malaria hotspot location paradigm as some urban-rural areas in the East region recorded new incident malaria hotspots for 2015 which was not seen in the previous years. In a study**(**32)**,** which focused on the mapping of *Plasmodium falciparum* mortality in Africa between 1990 to 2015, the authors reported that several malaria hotspots areas in Cameroon, Niger, Central Africa Republic and Ivory Coast, were associated with high mortality rate and low coverage of antimicrobial treatment(> 20 malaria deaths per 10,000) [32]. This study did not locate in detail, the various regions or urban-rural areas in Cameroon with such hotspots. Our hotspots maps are an affirming tool at the regional and even urban-rural scale for malaria prevention programs. Furthermore, [32] identified regions of Adamawa, North, and East of having high mortality (> 20 per 10,000) and low drug treatment < 10%. Our study reported these regions of having low malaria cases and no statically significant malaria hotspots except for the East region. However, our study focused on malaria cases and advise for continues preventive measures in the urban-rural areas or regions of low malaria cases and high mortality.

This study reported on the use of ITNs and IRS as one of the most effective preventive strategy for malaria control in Cameroon and though an effective method, Fig. 1 demonstrates low (< 50%) coverage of ITNs and or the use of IRS in all the regions. More campaigns and universal distribution of free ITNs that was initiated in Cameroon in 2011 [4] should be focused in urban-rural areas of regions with very low ITNS/ IRS usage. A study on the *Mapping of Plasmodium falciparum* mortality in Africa between 1990 and 2015 estimated that areas with high mortality rates(10–20 per 10,000) were associated with low coverage of ITNs (30–50%) for most regions in Cameroon, Nigeria, Angola and parts of Congo, Central African Republic, Guinea and Equatorial Guinea [32]. Furthermore, an observational study that assesed ITNs possesion and their protective effects on malaria infection in semi-urban and rural communities in the South West region of Cameroon, found that ITNs ownership was lower in rural settings compared to semi-urban settings [4]. This also calls for malaria prevention and control campaigns such as those on ITNs distributions in urban-rural areas and particularly hotspots locations.

The population density map (Fig. 8) at the urban-rural areas showed that malaria cases and hotspots locations were higher in regions of higher population density and lower in regions of lower population density. This corroborated with the findings of Kabaria [33] who reported the relationship between human population densities and malaria infection risk in children aged < 5 in Africa using the DHS data. They identified the correlation between high malaria risk prevalence in urban areas and argues for the decrease in transmission in rural areas due to urbanization. Yaoundé (Central region) and Douala (littoral region) are the capital and economic capital of Cameroon respectively and are full of more human activities than the other regions. We could not evidently support the reasons for the association between high malaria cases and high population densities and call for more research at a smaller scale in the future.

The Pearson’s coefficient, r (Table 3) shows a positive association with some environmental factors such as rainfall, vegetation, and nightlights. Again, this is not a new finding as a similar report on malaria prevalence on climatic factors have been demonstrated in Cameroon, where the authors derived spatial distribution maps for malaria transmission under different climatic and intervention scenarios. Their predictive study showed that temperature and rainfall were associated with malaria transmission [34]. The association between malaria cases and rainfall (*p* < 0.001 and *r* = 0.25) examined in 2015 for example, highlights the necessity for malaria surveillance and response systems during the rainy seasons in Cameroon since standing water provides breeding grounds for anopheles mosquitos responsible for transmission of the parasites. In the northern part of the country, the rainy seasons are from May to September (little rainfall) and from March to August (major rainfall) in the southern part. Moreover, the nightlights composite (p < 0.001 and *r* = 0.44) in 2015 which indicates the number of human activities at night shows that cities in Cameroon such as Douala and Yaoundé with the highest population densities have more night time activities due to increasing urbanization. The government should carry out more malaria preventive measures and campaigns in the urban-rural areas of these regions. Vegetation Indices are spectral shift of two or more bands designed to heighten the contribution of vegetation properties and allow reliable spatial and temporal inter-comparisons of terrestrial photosynthetic activity and leaf canopy structural changes [35]. Vegetations near human settlements increase the population of malaria vectors and thus transmission of malaria. Kar [36] in their study; a review of malaria transmission dynamics in forest ecosystem illustrated that forests serve as beds for malaria transmission as they provide favorable conditions such as vegetation cover, temperature, rainfall, and humidity for malaria transmission. In Cameroon, most rural settlements and villages are located within forest areas and prevention campaigns should be extended to such areas with malaria clusters and hotspots. Our study has the following limitations; i) The malaria prevalence clusters and hotspots at the various urban-rural areas, could be misinforming as the GPS clusters data for these areas were displaced for confidentiality, though the clusters were maintained within the DHS administrative unit. ii) our study did not use socio-demographic factors that could find the association between malaria prevalence and social determinants of health and some related environmental data were missing, iii) The DHS project samples collection are subjected to bias due to disparities in the different urban-rural settings and various forms of bias such as the interviewee response bias. iv) the correlation analyses may be confounded by other factors and spatial techniques such as the geographically weighted regression may be considered to analyze the association between environmental variables and malaria distribution. We did not apply this technique because of missing GPS urban-rural data points in some of the malaria years.

The strength of this study includes; the application of spatial statistics and the use of ArcGIS in malaria research at a smaller geographic scale for public health interventions, the design of this study demonstrated the importance of using spatial data in DHS research. Also, our study, unlike others will provide a new insight to the prevention of malaria in Cameroon at the small-area scale and the techniques used can be applied to other disease phenomena.

## Conclusion

This research focused on malaria distribution at a smaller scale (urban-rural) and we identified urban-rural areas with high and low malaria cases and hotspots. Global spatial demographic health datasets have been used to estimate the population at risk of malaria, which forms a fundamental system of measurement for decision-makers at national and international levels [37–39]. Our maps are supporting tools for effective malaria control at the urban-rural scale and can be used to inform malaria prevention and control programs. Despite the current advances to prevent malaria, more work is required particularly in targeting the population at the urban-rural geographic scale on spatial data collection and surveys, wide coverage and distribution of ITNs, campaigns, screening, and provision of treatment that will progressively eliminate the disease.

## Abbreviations

DHS: Demographic and Health Surveys
GIS: Geographical Information Systems
GPS: Global Positioning System
IRB: Institution Review Board
IRS: Indoor Residual Spray
ITNs: Insecticides treated bed nets
MI: Moran’s Index
MIS: Malaria Indicator Survey
PCC: Pearson’s Product-Moment Correlation Coefficient
RDT: Rapid Diagnostic Test
SARS: Severe Acute Respiratory Syndrome (SARS)
SSA: Sub-Saharan Africa
UN: United Nation
USAIDS: United States Agency for International Development
WHO: World Health Organization
